# Experimental and Theoretical Study of Multifrequency Surface Acoustic Wave Devices in a Single Si/SiO_2_/ZnO Piezoelectric Structure

**DOI:** 10.3390/s20051380

**Published:** 2020-03-03

**Authors:** Cinzia Caliendo, Farouk Laidoudi

**Affiliations:** 1 Institute for Photonics and Nanotechnologies, IFN-CNR, Via Cineto Romano 42, 00156 Rome, Italy; 2 Research Center in Industrial Technologies CRTI, P.O. Box 64 Cheraga, Algiers 16014, Algeria; f.laidoudi19@gmail.com

**Keywords:** ZnO, Rayleigh wave, Sezawa wave, single device structure, multifrequency device

## Abstract

The propagation of surface acoustic waves (SAWs) along a ZnO/SiO_2_/Si piezoelectric structure is experimentally and theoretically studied. Six surface acoustic modes were experimentally detected in the 134 to 570 MHz frequency range, for acoustic wavelength λ = 30 μm, and for SiO_2_ and ZnO layers with a thickness of 1 and 2.4 μm. The numerical and three-dimensional (3D) finite element method analysis revealed that the multilayered substrate supports the propagation of Rayleigh and Sezawa modes (R_m_ and S_m_), their third and fifth harmonics at λ/3 and λ/5. The velocity of all the modes was found in good agreement with the theoretically predicted values. Eigenfrequency, frequency domain, and time domain studies were performed to calculate the velocity, the electroacoustic coupling coefficient, the shape of the modes, the propagation loss, and the scattering parameter S_21_ of the SAW delay lines based on the propagation of these modes. The sensitivity to five different gases (dichloromethane, trichloromethane, carbontetrachloride, tetrachloroethylene, and trichloroethylene) was calculated under the hypothesis that the ZnO surface is covered by a polyisobutylene (PIB) layer 0.8 µm thick. The results show that the modes resonating at different frequencies exhibit different sensitivities toward the same gas. The multi-frequency ZnO/SiO_2_/Si single device structure is a promising solution for the development of a multiparameters sensing platform; multiple excitation frequencies with different sensing properties can allow the parallel analysis of the same gas with improved accuracy.

## 1. Introduction

The Rayleigh wave is a surface acoustic wave (SAW) that travels along the free surface of a piezoelectric half-space and its energy is concentrated up to about a depth of one wavelength. The wave is elliptically polarized and has three particle displacement components, U_1_, U_2_, and U_3_, that vanish at a depth of about one wavelength below the surface. The propagation of the SAW can be excited and detected by means of a couple of interdigitated metal electrodes (IDTs), the launching and the receiving IDT, placed onto the surface of the piezoelectric substrate. One IDT is connected to a radio frequency (rf) generator and converts the input rf signal into a mechanical deformation, the wave, showing the same periodicity of the IDT’s metal electrodes. The other IDT reveals the substrate deformation that converts into an electrical output signal. The wave velocity *v* = f·λ is determined by the wave frequency *f* and the SAW wavelength λ that is fixed and equal to the IDTs pitch [1]. 

In homogenous media, the confinement mechanism of the SAWs depends on the presence of a stress-free surface. For non-homogeneous materials, i.e., layered substrates, the waveguiding effect is induced by the layer and substrate elastic properties. If the layer loads the substrate, the acoustic waves within the layer are reflected from the substrate; this confinement gives rise to higher order modes that propagate under the restriction of finite penetration into the substrate. The number of these modes and their velocity depend on the layer thickness h: for a very thin film thickness (h/λ << 1), only the fundamental Rayleigh mode (R_m_) propagates with a velocity very close to the SAW velocity of the substrate material; by increasing the layer thickness (h/λ >> 1), the R_m_ velocity asymptotically reaches the SAW velocity of the layer material. The second order Rayleigh mode is generally called the Sezawa mode (S_m_) and the other modes are simply called R3, R4, and so on. The amplitude profile of R_m_ is predominantly confined in the layer and decays exponentially with the depth, while that of the higher order modes has an exponential tail in the substrate. The latter modes have a layer thickness-to-wavelength cut-off at which the phase velocity is equal to the substrate shear velocity. Right at the cut-off, the SAW mode couples with bulk modes and shows a leaky nature, as the acoustic power flows into the bulk substrate, thus, resulting in a large insertion loss. By increasing the layer thickness, the velocity of the higher order modes asymptotically reaches the shear velocity of the layer [2]. 

The electroacoustic devices based on the thin piezoelectric films technology have many advantages over the counterparts implemented on bulk piezoelectric materials, such as integration of the devices with electronics for control and signal processing, the capacity to control the device’s sensitivity to temperature changes; and fabrication of multisensor arrays that are able to provide multiple parallel sensing functions. ZnO is a wide bandgap semiconducting piezoelectric material that can be deposited in a thin film form using the sputtering technique onto non piezoelectric materials, such as Si. Moreover, thermally compensated multilayered electroacoustic devices can be designed if ZnO is combined with layer materials showing the opposite sign temperature coefficient of frequency [3,4]. ZnO has attractive piezoelectric and optical properties that make it suitable for use in several sensing applications including detection of UV light [5,6] and gas (NO_2_, ethanol, ammonia, to cite just a few) [7,8,9,10]. Because of its wide bandgap (3.4 eV), ZnO can recover the double role of piezoelectric medium and UV light absorber inside solar-blind UV SAW sensors.

In this study, we fabricated SAW delay lines on the surface of a thin ZnO layer sputtered onto the thermally oxidized surface of a Si (001) wafer; the delay lines consist of two interdigital transducers (IDTs) with a periodicity of 30 μm, which represents the acoustic wavelength λ. The SAW modes traveling in the ZnO/SiO_2_/Si < 100 > (001) structures were investigated both experimentally and theoretically. Six modes were observed: R_m_, S_m_, and their third and fifth overtones. The numerical calculation and three-dimensional (3D) FEM analysis were performed to study the modes shape, velocity, and propagation loss, and to assign each frequency that was experimentally observed the proper SAW mode. In layered structures, the phase velocity of each supported mode is dispersive, thus, the overtone frequencies are not simply proportional to the fundamental one, as opposed to the bulk acoustic wave-based devices. The experimental data were found in good agreement with the theoretically predicted values. The last paragraph shows the simulation of the gas sensing performances of the four modes to five different gases (dichloromethane, trichloromethane, carbontetrachloride, tetrachloroethylene, and trichloroethylene), under the hypothesis that the ZnO surface is covered by a PIB layer 0.8 µm thick.

## 2. Experimental Results

**Figure 1:**
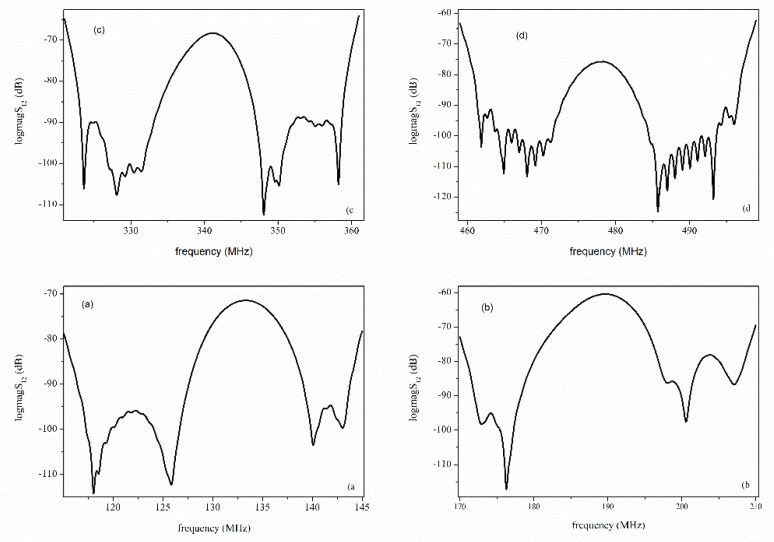
The scattering parameter S_21_ vs. frequency curves of (**a**) R_m_, (**b**) S_m_, (**c**) the R_m_ third harmonic, (**d**) the S_m_ third harmonic.

A c-axis oriented ZnO layer, 2.4 μm thick, was grown on a thermally oxidized Si(001) substrate using the rf sputtering technique at the following deposition conditions: rf power 200 watt, temperature 200 °C, O_2_/Ar atmosphere, pressure 3.7 × 10^−3^ Torr, and 99.99% pure Zn target. The SiO_2_ layer, 1 μm thick, was thermally grown on the Si substrate at the temperature T = 1012 °C for 4 h in a flux of wet O_2_ equal to 1 L/min. A Cr/Al layer (0.05/0.1 μm) was deposited onto the ZnO film in order to implement the SAW delay lines (SDLs) by a conventional photolithographic technique. The SDLs consisted of two IDTs with λ = 30 μm, N = 15 finger pairs, fingers overlapping w = 40·λ, and IDTs center-to-center distance L = 6.25 mm. The device’s backside was fixed to a TO8 test fixture and the IDTs pads were bonded by ultrasonic bonder to the TO8 connections. The device’s scattering parameter S_21_ was tested in the time and frequency domain with a vector network analyzer (VNA) HP 8753A connected to a data acquisition system; the VNA was calibrated with handmade calibration standards. The direct electromagnetic coupling between the IDTs was eliminated using the gating technique. The modes observed were the following: the R_m_ at about *f*_Rm_ = 133.6 MHz and the S_m_ at *f*_Sm_ = 189 MHz. The modes’ velocities were *v*_Rm_ = *f*_Rm_ λ = 4008 m/s and *v*_Sm_ = *f*_Sm_ λ = 5670 m/s, respectively, for λ = 30 μm, the wavelength was most effectively excited by the IDT as it is equal to the periodicity of the transducer pattern. The S_m_ had a much higher acoustic velocity and larger signal amplitude than that of the R_m_ wave, which indicated that the electromechanical coupling constant of this mode was higher than that of the R_m_. The Rayleigh third and fifth harmonics were observed at 340 and 400 MHz and the Sezawa third and fifth harmonics were observed at 480 and 570 MHz; while the third harmonic signals were as high as that of the corresponding fundamental mode, the fifth harmonic signals were clearly visible but weak. Figure 1 shows the scattering parameter |S_21_| vs. frequency curves of the fundamental and third harmonic modes.

The assignment of the measured resonances to the SAW modes was confirmed by finite element method (FEM) calculations, using COMSOL 5.4 and Ansys software, and discussed in the following paragraphs. Eigenfrequency and frequency domain analysis were used to determine the resonance frequencies and the modes shape in the multilayer system. Time domain analysis was used to simulate the S_21_ vs. frequency curves. The material parameters used in the calculations are given in reference [11,12] for Si and SiO_2_, while the ZnO single crystal and thin film material constants are those from references [12,13].

**Figure 2:**
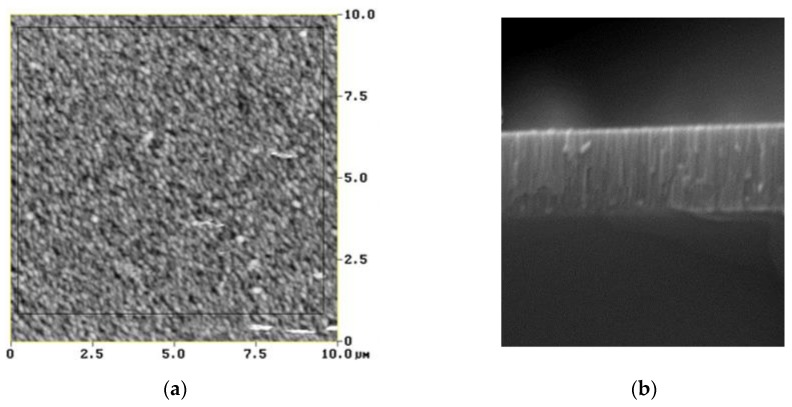
(**a**) The atomic force microscopy (AFM) and (**b**) the scanning electron microscopy (SEM) photo of the ZnO film.

Atomic force microscopy (AFM) measurements (Figure 2a) were conducted to study the surface morphology of the ZnO films. The root mean square (RMS) surface roughness value for ZnO films with the same thickness and grown in different sputtering runs was measured and the result was equal to approximately 8 nm. 

Figure 2b shows the scanning electron microscopy (SEM) image of the cross section of the ZnO film. The crystalline growth of the ZnO layer is evident in the cross-section SEM image: the piezoelectric film contains a columnar structure and the growth direction of the columns is perpendicular to the sample surface.

The piezoelectric strain constant, d_33_, of the ZnO film grown on the metallized surface of the Si substrate was measured by following the method described in reference [14] and based on the direct piezoelectric effect: a longitudinal acoustic wave perturbs the sample and the electrical voltage induced in the piezoelectric film is measured [15]. The probe consisted of a metal rod in contact with a Pb(Zr,Ti)O_3_ (PZT)-based low frequency (2 MHz) transducer that was connected to a pulse generator (pulse width 0.1 to 1.0 ns) to produce longitudinal bursts propagating along the metal rod. The contact between the rod and the piezoelectric film surface resulted in the application of a stress on the surface of the ZnO film. Stress-induced electrical charges collected at the ZnO film surfaces were observed on an oscilloscope. The d_33_ of the tested films was evaluated by comparing the response signal with that obtained on a standard piezoelectric thin plate in the same conditions, by following the procedure outlined in reference [16]. 

All the tested films showed to be piezoelectric with a difference in the d_33_ obtained values not appreciable with this measurement technique because of an error of about 15% to 20%. The estimated mean value is 9 pC/N and this value is in good agreement with the corresponding value of approximately 12 pC/N, reported in the available literature [17].

The crystalline quality of the ZnO film was investigated by X-ray diffraction (XRD) analysis on a Rigaku diffractometer in the Bragg–Brentano geometry using the Cu Kα line (λ = 1.5418 Å), with diffracted intensities collected in a θ-2θ scan mode. The diffraction patterns showed only a strong peak at 2θ = 34.30° which indicates that the films are highly c-axis oriented and have wurtzite crystal structure. The full width at half maximum (FWHM), equal to about 0.3°, indicates that any misfit strain in the films is completely relaxed.

## 3. Theoretical Study

### 3.1. Numerical Calculations

**Figure 3:**
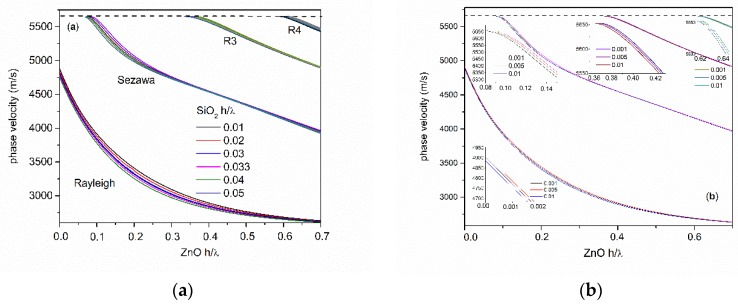
The phase velocity dispersion curves of R_m_, S_m_, R3, and R4 modes for SiO_2_ thickness-to-wavelength ratio ranging (**a**) from 0.01 to 0.05 and (**b**) from 0.001 to 0.01.

The phase velocity dispersion curves of the surface acoustic waves traveling along the surface of the ZnO/SiO_2_/Si structure were numerically calculated by using McGill software [18], assuming that the surface of the ZnO layer is electrically and mechanically free. The material constants used in the calculations (mass density, elastic, dielectric, and piezoelectric constants) were extracted from reference [12] and are referred to single crystal materials. The presence of the metal IDTs was not taken into account in the numerical calculations, all the materials were assumed lossless. Figure 3a,b shows the dispersion curves of the first four Rayleigh modes calculated for different SiO_2_ layer thickness values, from 0.001·λ to 0.05·λ.

The phase velocities of the Sezawa and higher order modes have a high frequency asymptote corresponding to the shear velocity of the ZnO layer, as opposed to the Rayleigh wave which has an asymptote equal to the SAW in the ZnO layer. Sezawa and the higher order SAW modes have a cut-off thickness-to-wavelength ratio at which the phase velocity is equal to the substrate shear velocity [2]; the existence of those modes is only possible for ZnO values ≥ h/λ_cut off_. By increasing the SiO_2_ layer thickness (from 0.001·λ to 0.05·λ), the cut-off of the S_m_, R3, and R4 modes decreases; it ranges from 0.098·λ to 0.071·λ for S_m_, from 0.3698·λ to 0.334·λ for R3, and from 0.6169·λ to 0.5887·λ for R4. The SAW modes in this structure are dispersive, and their velocities are strongly related to the materials properties, thickness of each layer, substrate crystal cut and propagation direction. The presence of the SiO_2_ layer between the Si substrate and the piezoelectric ZnO layer, although not essential in the operation of the device, however, plays useful roles. The SiO_2_ layer electrically isolates the Si substrate and allows field penetration in it [19], and it improves the growth of high quality c-axis oriented ZnO films. Depending on the ZnO thickness-to-wavelength ratio, the ZnO film thickness required for a given resonant frequency can be lower for the ZnO/SiO_2_/Si structure than for the ZnO/Si one [20]. Moreover, as ZnO and Si have a positive temperature coefficient of delay (TCD), whereas SiO_2_ has a negative TCD, it is possible to design a temperature compensated ZnO/SiO_2_/Si-based device at a specific frequency [3,21].

**Figure 4:**
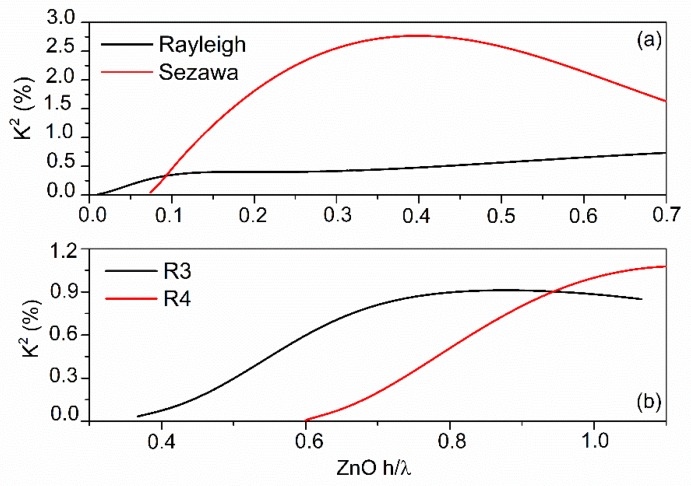
The K^2^ dispersion curves of the first four modes for SiO_2_ h = 0.033·λ.

The electroacoustic coefficient K^2^ is a measure of the percentage of electrical-to-acoustic transduction efficiency of the IDTs. K^2^ can be calculated as K^2^ = 2∙((v_free_ − v_met_)/v_free_ ) where v_free_ and v_met_ are the velocity of the SAW traveling along the electrically free and short-circuited surface of the ZnO layer. Figure 4 shows the K^2^ dispersion curves of the first four modes for SiO_2_ h/λ = 0.033. The multilayered structure also supports the propagation of Love modes (LM) but these waves are not electrically active as their K^2^ is equal to zero. It can be observed that the K^2^ of the S_m_ reaches a maximum value (2.77%) at h/λ = 0.4, then it decreases but maintains values larger than the other modes for h/λ values up to 0.7. The K^2^ dispersion curve of R_m_ increases slightly with 1/λ and stabilizes at small λ. The largest K^2^ values correspond to the R_m_ and S_m_ for ZnO thickness up to approximately 0.63·λ. 

According to the theoretical calculations, only the Rayleigh and Sezawa modes can be excited for SiO_2_ and ZnO normalized thickness h/λ = 0.033 and 0.08, for λ = 30 μm. A rough prediction of the expected harmonic frequencies can be obtained from the dispersion curves by selecting, on these curves, the wave velocity corresponding to the harmonic wavelength [22]. Possible fluctuations of the films’ thickness can reduce the accuracy in the estimation of the harmonic frequencies.

### 3.2. 3D FEM Analysis: Eigenfrequency Study

**Figure 5:**
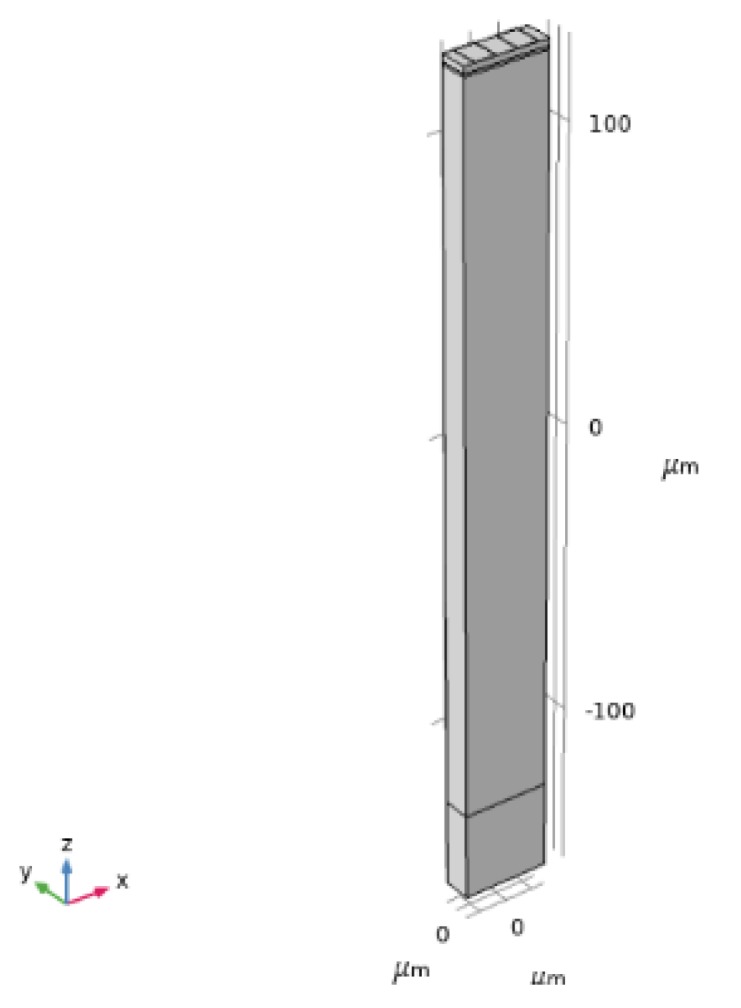
The three-dimensional (3D) primitive cell.

The propagation of surface acoustic waves along the Si/SiO_2_/ZnO structure was investigated by using 3D FEM analysis. Comsol Multiphysics 5.4 software was used in the eigenfrequency study. The model uses a piezoelectric multiphysics coupling node with solid mechanics and electrostatic interfaces. The primitive 3D cell, shown in Figure 5, is one wavelength wide, with a depth equal to λ/3; it has two periodic boundary conditions applied on the sides, in solid mechanics and in the electrostatics module. A perfectly matched layer (PML), one λ in height, was added to the bottom of the silicon substrate, in order to prevent waves reflection from the Si bottom. In these specific simulations, the wavelength is λ = 30 µm and the thickness-to-wavelength ratio of the layer materials was set to h_SiO2_/λ = 0.033, and h_ZnO_/λ = 0.08. The simulations accounted for two Al IDT fingers, 0.1 μm thick and λ/4 wide, located on the free surface of the ZnO layer. The terminal (1V) and ground electrical boundary conditions were applied at the interdigitated electrodes. The silicon substrate was assumed to be anisotropic and the propagation direction was assumed to be along the x-axis on the z-plane; the silicon thickness was assumed equal to 250 μm only as the mechanical displacement depth of the wave modes are most confined at the surface and nearly die out at a lower boundary. ZnO was assumed to be a single crystal material.

**Figure 6:**
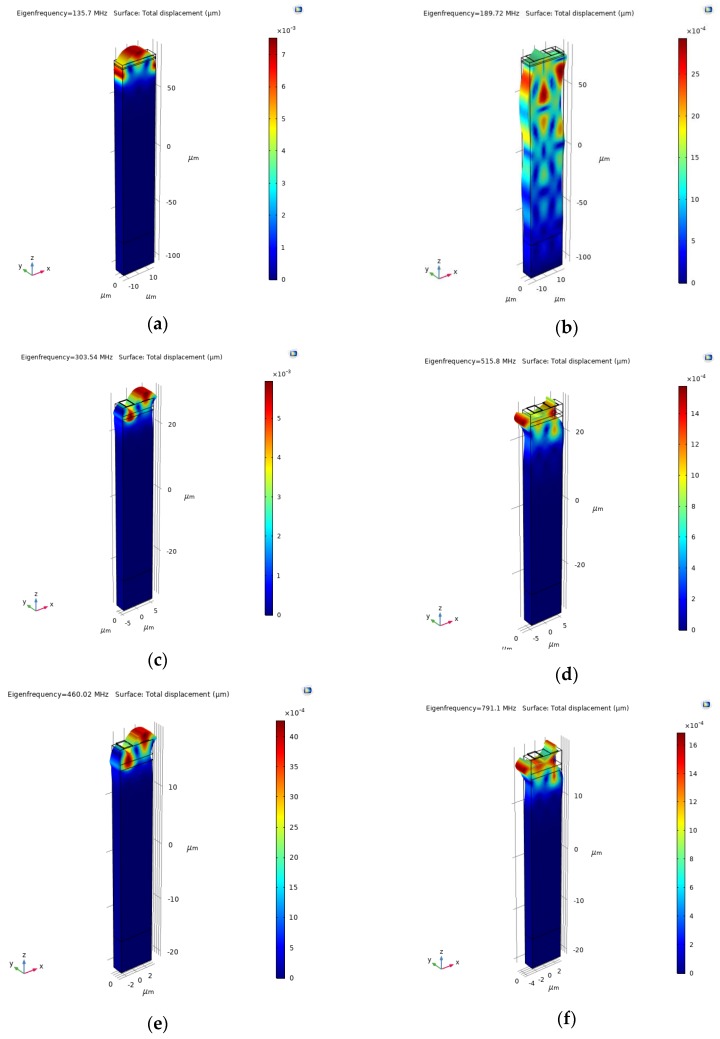
The solid displacement of R_m_ and S_m_, for λ = 30 μm (**a**,**b**); for λ = 10 μm (**c**,**d**); and for λ = 6 μm (**e**,**f**).

The ZnO and SiO_2_ were assumed to have an elastic and dielectric loss tanδ = 0.002 and 0.01. Figure 6a–f shows the absolute total displacement of R_m_ and S_m_ obtained by eigenfrequency analysis, for λ = 30, 10, and 6 µm. 

The R_m_ and S_m_ resonant frequencies are quite sensitive to the ZnO material constants used in the calculations, especially the harmonic modes. The modes resonant frequencies were calculated by using both the single crystal and the polycrystal ZnO material constants. For λ = 30, 10 and 6 µm, the Rayleigh mode frequencies are 135.6, 303.54, and 460.02 for the ZnO single crystal and 132.6, 296.5, and 480 MHz for the ZnO film. The Sezawa mode frequencies are 189.72, 515.8, and 791.1 for the ZnO single crystal and 188.45, 480, and 745 MHz for the ZnO film. The theoretical frequencies show a little discrepancy with the experimental ones; the discrepancy can be attributed to the accuracy in the measurement of the films’ thicknesses, as well as to the layers’ material constants used in the calculations.

The 3D eigenfrequency study accounts for the three particle displacement components of the waves, U_1_, U_2_, and U_3_ that are parallel to the x-, y-, and z-axis of the reference system shown in Figure 4, Figure 5 and Figure 6. Since the shear horizontal (U_2_) component is much smaller that the longitudinal (U_1_) and shear vertical (U_3_) displacement components, then a two-dimensional (2D) frequency domain study (which account for only U_1_ and U_3_) is sufficient to study the SAW modes in c-ZnO/SiO_2_/Si < 100 > (001) structures. 

### 3.3. 2D FEM Analysis: Frequency Domain Study

**Figure 7:**
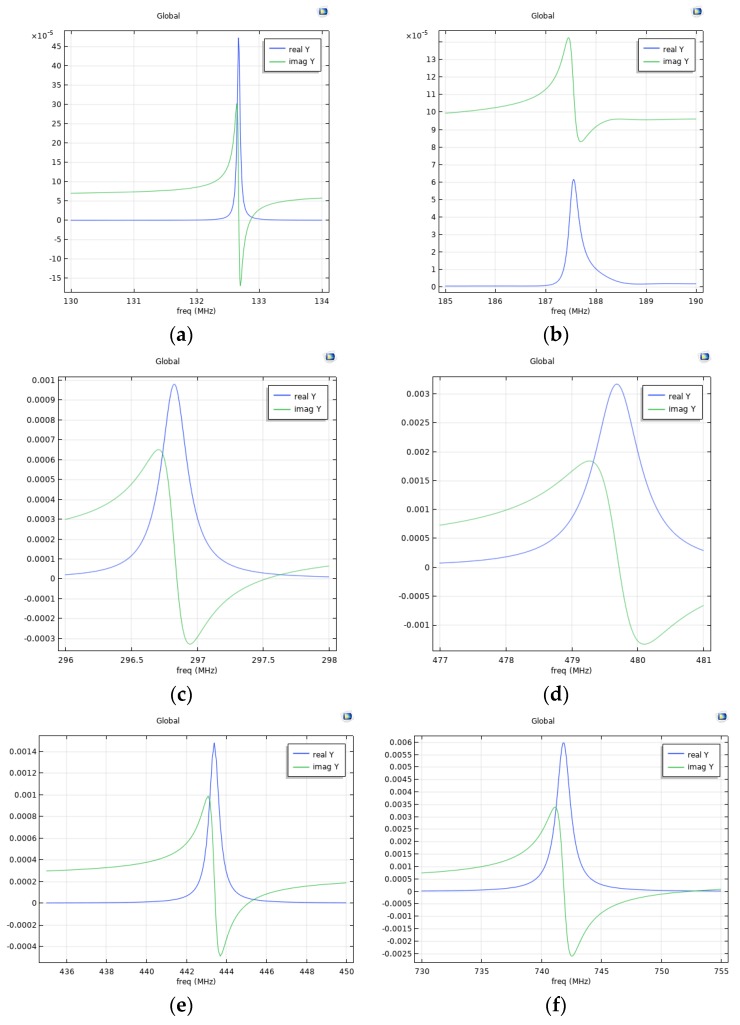
The real and imaginary part of the admittance Y vs. frequency curves showing the resonance peaks of the (**a**) R_m_ and (**b**) S_m_ calculated for λ = 30 μm; (**c**) R_m_ and (**d**) S_m_ calculated at 10 μm wavelength; (**e**) R_m_ and (**f**) S_m_ calculated at 6 µm wavelength.

The 2D frequency domain study was performed by Comsol 5.4 software to evaluate the resonance frequency and the K^2^ of the SAWs traveling along the Si/SiO_2_/ZnO structure through the calculation of the admittance vs. frequency curves. The simulations accounted for two Al IDT fingers, 0.1 μm thick and λ/4 wide, located on the free surface of the ZnO layer. The terminal (1 V) and ground electrical boundary conditions were applied at the interdigitated electrodes. The ZnO and SiO_2_ were assumed to have an elastic and dielectric loss tanδ = 0.002 and 0.01. In these specific simulations λ = 30 µm resulted in an IDTs pitch of p = 15 µm; the Al strip width to spacing ratio was fixed to 1. A perfectly matched layer (PML), one wavelength wide and thick, was added to the bottom of the Si substrate, 5·λ thick, to avoid reflections from the bottom side of Si; periodic boundary conditions were applied to the left and right sides of the structure; the continuity of displacements and electric field at the interfaces level is automatically activated in COMSOL environment. Figure 7a–f shows the real and imaginary parts of the electrical admittance Y vs. frequency curves showing the resonance peaks of the fundamental, third and fifth harmonics of R_m_ and S_m_.

The K^2^ values of the acoustic modes were evaluated according to the formula K2=π24·[(fp−fs)fs], where *f_p_* and *f_s_* are the series and parallel resonance frequency. The following K^2^ values were obtained for the R_m_ and S_m_: 0.19% and 0.39% for λ = 30 μm, 0.25% and 0.41% for the third harmonics (λ = 10 μm), and 0.33% and 0.47% for the fifth harmonic modes (λ = 6 μm). 

The calculated frequencies are in good accordance with the experimental values. A small deviation between experiment and simulation results could be attributed to the uncertainties in the layers’ thicknesses and material constants. 

### 3.4. 3D FEM Analysis: Time Domain Study

**Figure 8:**
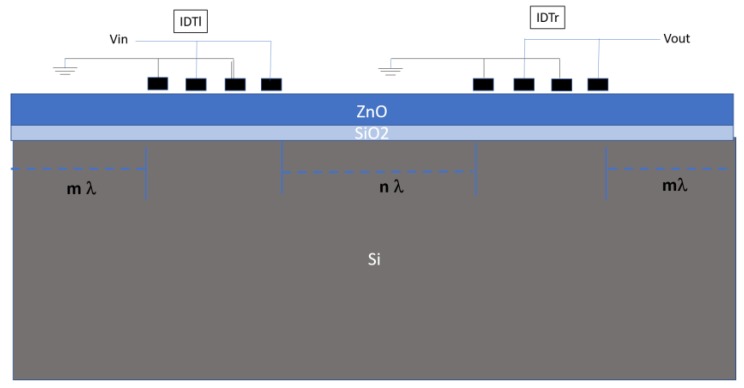
The schematic of the delay line model adopted for the time domain study.

A 3D simulation of a SAW delay line was performed in the time domain by Ansys software. The model includes a thin piezoelectric layer of ZnO, 2.4 µm thick; an isotropic SiO_2_ layer, 1µm thick; and a Si (001) substrate 150 µm thick. The ZnO and SiO_2_ elasticity and dielectric losses were assumed equal to 0.002 and 0.01. The IDT’s are modeled by two finger pairs with fingers separated by λ/4 from each other, as shown in Figure 8, where m·λ represents the distance between the substrate vertical edge and the IDT finger, n·λ represents the distance between the two IDTs, m and n are equal to 10 and 3 for the fundamental and third harmonic modes at λ = 10 µm, and are equal to 10 and 2 for Rayleigh and Sezawa modes at λ = 30 µm. This choice was done to decrease the calculations time. The nodes at the bottom of the structure were constrained (U_x_ = U_y_ = 0). Continuity boundary conditions were applied on the interfaces ZnO/SiO_2_ and SiO_2_/Si to ensure the continuity of displacements and electric field between the layers. Two electrodes of both the launching and receiving IDT (IDTl and IDTr) were addressed with ground potential (V = 0 V); the other two electrodes of the IDTl were addressed with a harmonic time dependent potential V_input_ = V_0_∙cos(2∙π∙f_0_∙*t*) with a voltage amplitude of V_0_ = 1 V peak; the time duration *t* of the V_input_ is defined in order to prevent reflections from the free end sides of the model. The frequency of the excitation f_0_, determined by the eigenfrequency study, is referred to the mode under study. The output waveform is recorded as a voltage signal V_out_ at the electrodes of the IDTr. A time dependent solver was chosen with discrete time steps of 1/512 of the oscillation period for a time range of six to 10 wave cycles, depending on the mode studied.

**Figure 9:**
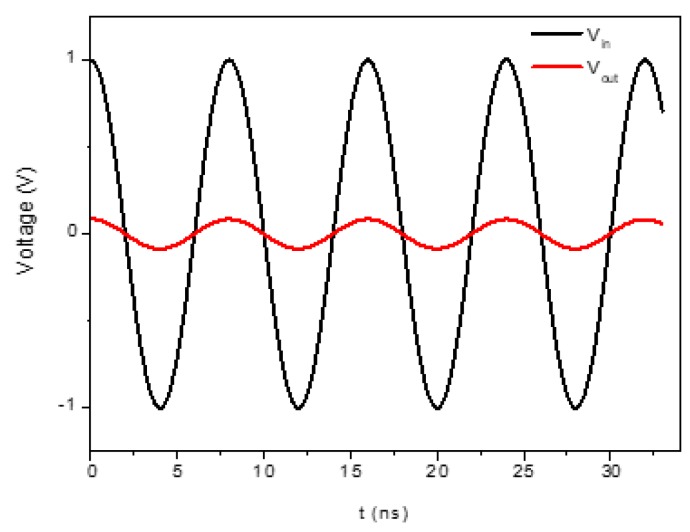
The input and output voltage vs. time curves for the Rayleigh mode.

Figure 9 shows, as an example, the input and output voltage vs. time curves referred to the R_m_. As expected, the amplitude of the output waveform is shorter than that of the applied input as a consequence of the energy loss due to the SAW decay in the substrate depth. The application of V_input_ to the IDTl gives a stable sinusoidal output of amplitude 0.0848 V at the IDTr.

The frequency dependence of the scattering parameter S_21_ of the delay line was calculated by making the time-to-frequency fast Fourier transform (FFT) of V_input_ and V_output_ by Matlab, and then applying the formula S_21_ = 20∙log (V_in_/V_out_). 

**Figure 10:**
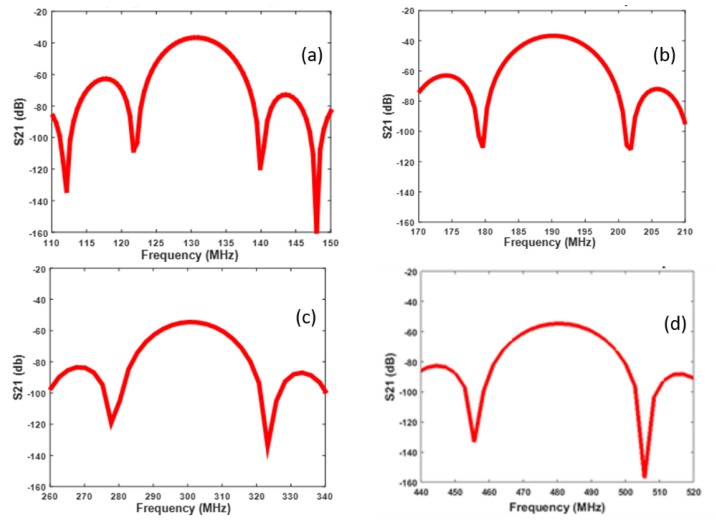
The S_21_ of the fundamental (**a**) Rayleigh; (**b**) Sezawa; and third harmonic of the (**c**) Rayleigh; and (**d**) Sezawa mode vs. frequency.

Figure 10 shows scattering parameter S_21_ vs. frequency curves of the fundamental and third harmonic of the R_m_, S_m_ guided mode-based delay lines. The bandwidth of the latter modes is larger than that of the R_m_ and S_m_, since their acoustic energy is mostly trapped in the ZnO/SiO_2_ bilayer, while the former modes energy decays for some wavelengths in the depth of the structure. The insertion loss of the two harmonics was larger than that of the R_m_ and S_m_, in accordance with the experimental data. The calculated propagation loss was obtained as $\frac{S_{21}\left(dB\right)}{n\cdot \lambda }$, being *n·λ* the distance between the two IDTs: 0.61 dB/µm (1.22 dB/λ) for R_m_ and S_m_ while it is 1.65 dB/µm (4.95 dB/λ) and 1.87 dB/µm (5.61 dB/λ) for the third harmonic of the R_m_ and S_m_, respectively.

## 4. Gas Sensing Simulation

**Figure 11:**
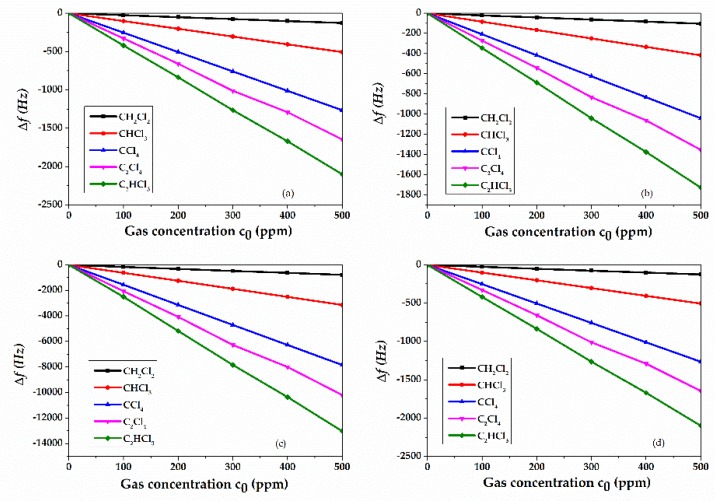
The frequency shift vs. gas concentration curves of the (**a**) Rayleigh and (**b**) Sezawa; the third harmonic of (**c**) R_m_ and (**d**) S_m_.

The behavior of the Si/SiO_2_/ZnO SAW devices operating as gas sensors was studied by 2D FEM with Ansys software, under the hypothesis that the surface of the ZnO layer is covered with a thin polyisobutylene (PIB) film, 0.8 µm thick. The sensor was investigated for the detection of the following five volatile organic compounds at atmospheric pressure and room temperature: dichloromethane (CH_2_Cl_2_), trichloromethane (CHCl_3_), carbontetrachloride (CCl_4_), tetrachloroethylene (C_2_Cl_4_), and trichloroethylene (C_2_HCl_3_) [23,24]. The PIB interaction with the gas molecules, i.e., the sensing mechanism, was simulated as an increase in mass density, ρ = ρ_unp_ + ∆ρ, being ρ_unp_ the unperturbed mass density of the PIB layer (in air) and ∆ρ the partial density of the gas molecules adsorbed in the PIB layer, ∆ρ = K·M·c_0_·P/RT, where P and T are the ambient pressure and temperature (1 atm and 25 °C), c_0_ is the gas concentration in ppm, K = 101.4821 is the air/PIB partition coefficient for the studied gas, M is the molar mass, and R is the gas constant [24,25,26,27]. Any effects of the gas adsorption on the PIB layer properties other than the density changes were neglected. The PIB gas adsorption was simulated for gas concentration c_0_ in the range from 100 to 500 ppm. The mechanical properties of the PIB layer and the physical properties of the volatile gases were obtained from references [28,29]. It was found that the resonance frequencies of the four modes were downshifted by the adsorption of the gas into the PIB layer. The adsorbed gas increased the PIB mass density and lowered the phase velocity (and then the operating frequency), which can be correlated to the gas concentration. Figure 11a–d shows the resonant frequency shift vs. gas concentration for the fundamental and third harmonic R_m_ and S_m_.

The frequency shift of each mode, Δf = fair − fc_0_, being f_air_ and fc_0_ the resonant frequency values in air and at gas concentration c_0_, increases linearly with respect to the gas concentrations c_0_. The slope of the curves (the frequency shift per unit gas concentration, i.e., the sensor sensitivity S_c0_) increases with increasing the resonant frequency. Table 1 lists the frequency shifts per unit gas concentration of each mode. With decreasing λ, the resonant frequency and the sensors sensitivity increase. The gas sensitivities of R_m_ and S_m_ are quite similar at λ = 30 µm, while at λ = 10 µm the S_m_ sensitivity is more than twice that of the R_m_. For the same type of gas, the modes have a different sensitivity; each mode has a different sensitivity to each type of gas.

The sensor resolution, SR, is a measure of the minimum change of the input quantity to which the sensor can respond. Here, the limit of gas concentration resolution was assumed to be the c_0_ value that causes a frequency shift three times 1 Hz, SR = 3/S_c0_ [10]. Table 1 lists the SR values of the four modes for five different type of gases.

In reference [23] the gas sensitivity to CHCl_3_, CCl_4_, C_2_HCl_3_, and C_2_Cl_4_ of Rayleigh and Sezawa modes in PIB(110 nm)/AlN/SiO_2_/Si structure (λ = 4 µm) is theoretically investigated in the 1 to 10 ppm gas concentration range. The resonant frequencies are 1.169 GHz for the Rayleigh mode and 1.207 GHz for the Sezawa mode. The sensors showed a sensitivity that ranges from 0.75 to 12 Hz/ppm for the Rayleigh mode, and from 1.57 to 25 Hz/ppm for the Sezawa mode. These sensitivity values are lower than those referred to our structure for both the Rayleigh and Sezawa modes at λ = 10 µm; moreover, our structure is based on fixed ZnO and SiO_2_ layers’ thicknesses, as opposed to the case described in reference [23] where different AlN and SiO_2_ layers’ thicknesses are required to excite the Rayleigh wave (both AlN and SiO_2_ are 2 µm thick) and the Sezawa wave (AlN and SiO_2_ are 2 and 3 µm thick).

In reference [30], the sensitivity to six volatile organic gases (chloromethane, dichloromethane, trichloromethane, carbontetrachloride, tetrachloroethene, and trichloroethylene) at fixed concentration (100 ppm) is theoretically calculated for a SAW sensor implemented on a yz-LiNbO_3_ piezoelectric substrate covered by a PIB layer, 0.5 µm thick, with operating frequency f_0_ = 1.126 GHz, being λ = 3 µm. The response (frequency shift) of this sensor to 100 ppm gas concentration ranges from 25 to 21831 Hz, and corresponds to a relative frequency shift Δf/f_0_ = −0.02 and −19 ppm. These Δf/f_0_ values are comparable with those from our sensor whose Δf/f_0_ ranges from −0.11 to −3 ppm for R_m_ and S_m_ excited by λ = 30 µm, and from −0.52 to −12 ppm for the two modes excited by λ = 10 µm.

The present simulation results show that the four modes exhibit different sensitivities toward the same gas as well as different detection limit. Thus, each SAW mode can be addressed to the detection of a specific target analyte, while multiple detection of the same gas performed with four different sensibilities allows increased accuracy of the gas concentration measurement. 

## 5. Conclusions

Multimode acoustic wave devices were successfully fabricated on a ZnO/SiO_2_/Si structure. Six surface acoustic modes were experimentally detected in the 135 to 570 MHz frequency range, for acoustic wavelength λ = 30 μm, and for SiO_2_ and ZnO layers’ thicknesses of 1 and 2.4 μm. The propagation of SAWs along a ZnO/SiO_2_/Si piezoelectric structure was theoretically studied to investigate the nature of the traveling modes. Numerical and 3D FEM analysis revealed that the multilayered substrate supports the propagation of the Rayleigh and Sezawa modes, as well as their third and fifth overtones excited by λ = 10 and 6 μm. The small discrepancies between theoretical results and experimental ones can be attributed to some effects, including nonuniformity in the thickness of the ZnO layer and deviations between material constants used in the calculations. Eigenfrequency, frequency domain, and time domain studies were performed to calculate the velocity, the electroacoustic coupling coefficient, and the shape of the modes, and the scattering parameter S_21_ of the SAW delay lines based on the propagation of these modes. The sensitivity to five different gases (dichloromethane, trichloromethane, carbontetrachloride, tetrachloroethylene, and trichloroethylene) was calculated under the hypothesis that the device surface is covered by a PIB layer, 0.8 µm thick. The results show that the modes resonating at different frequencies exhibit different sensitivities toward the same gas, as well as different detection limits; higher sensor frequencies are advantageous as the sensitivity increases with the frequency. Thus, each SAW mode can be addressed to the detection of a specific target analyte to obtain a quantitative characterization of the surrounding environment. In addition, multiple detection of the same gas performed with four different sensibilities allows increased accuracy of the gas concentration measurement. Compared to the multimode devices based on the propagation of higher order SAW modes, which corresponds to a decreasing K^2^ value with the order of the mode, the present device offers the advantage of having K^2^ as high as those corresponding to the fundamental R_m_ and S_m_.

The results demonstrate that the Si/SiO_2_/ZnO layered structure is a promising solution to design high frequency SAW devices without the need of costly nanofabrication techniques. Moreover, the multimode operation in a Si/SiO_2_/ZnO single device structure is potentially attractive for application in the field of multicomponent gas analysis, as well as for developing a multiparameter sensing platform for UV light detection, temperature, and relative humidity measurement, to cite just a few. The sensor system also shows the inherent advantage to be suitable for passive and wireless operation; moreover, its architecture can include the integration of the surrounding electrical circuits. 

In the future, this system could be applied to UV sensing, and different modes could be screened for optimal sensing performances. Preliminary tests for UV sensing have been successfully performed. 

## Figures and Tables

**Table 1:** The frequency shift Δf per unit gas concentration and the SR of the four SAW modes for different gases, at a fixed PIB layer thickness (0.8 µm).

gas	Δf/Δc_0_ (Hz/ppm)				SR (ppm)			
	λ = 30 µm		λ = 10 µm		λ = 30 µm		λ = 10 µm	
	R_m_	S_m_	R_m_	S_m_	R_m_	S_m_	R_m_	S_m_
CH_2_Cl_2_	−0.25	−0.21	−1.57	−3.52	−11.86	−14.48	−1.91	−0.85
CHCl_3_	−1.01	−0.83	−6.27	−14.07	−2.96	−3.61	−0.48	−0.21
CCl_4_	−2.53	−2.08	−15.69	−35.17	−1.19	−1.44	−0.19	−0.09
C_2_Cl_4_	−3.28	−2.70	−20.37	−45.63	−0.91	−1.11	−0.15	−0.07
C_2_HCl_3_	−4.19	−3.45	−26.00	−58.24	−0.72	−0.87	−0.12	−0.05

## References

[1] Ruppel C.C., Fjeldly T.A. (2001). Selected Topics in Electronics and Systems: Volume 20.

[2] Mason W.P. (1972). Physical Acoustics: Principles and Methods.

[3] Emanetoglu N.W., Patounakis G., Muthukumar S., Lu Y. Analysis of Temperature Compensated SAW Modes in ZnO/SiO_2_/Si Multilayer Structures. Proceedings of the IEEE Ultrasonics.

[4] Martin S.J., Schwartz S.S., Gunshor R.L., Pierret R.F. (1983). Surface Acoustic Wave Resonators on A Zno-On-Si Layered Medium. J. Appl. Phys..

[5] Kumar S., Kim G.H., Sreenivas K., Tandon R.P. (2009). Tandon, Zno Based Surface Acoustic Wave Ultraviolet Photo Sensor. J. Electroceram..

[6] Emanetoglu N.W., Zhu J., Chen Y., Zhong J., Chen Y.M., Lu Y. (2004). Surface Acoustic Wave Ultraviolet Photodetectors Using Epitaxial Zno Multilayers Grown on R-Plane Sapphire. Appl. Phys. Lett..

[7] Rana L., Gupta R., Tomar M., Gupta V. (2017). Zno/ST-Quartz SAW Resonator: An Efficient NO2 Gas Sensor. Sens. Actuators B Chem..

[8] Tasaltin C., Ebeoglu M.A., Ozturk Z.Z. (2012). Acoustoelectric Effect on the Responses of SAW Sensors Coated with Electrospun Zno Nanostructured Thin Film. Sensors.

[9] Raj V.B., Singh H., Nimal A.T., Sharma M.U., Tomar M., Gupta V. (2017). Distinct Detection of Liquor Ammonia by Zno/SAW Sensor: Study of Complete Sensing Mechanism. Sens. Actuators B Chem..

[10] Ballantine D.S., White R.M., Martin S.J., Ricco A.J., Zellers E.T., Frye G.C., Wohltjen H. (1996). Acoustic Wave Sensors: Theory, Design, & Physico-Chemical Applications.

[11] Slobodnik A.J., Conway E.D., Delmonico R.T. (1973). Air Force Cambridge Research Laboratories.

[12] Hellwege K.-H., Hellwege A.M. (1979). Landolt-Börnstein, Numerical Data and Functional Relationships in Science and Technology, New Series, Group III.

[13] Carlotti G., Socino G., Petri A., Verona E. Elastic Constants of Sputtered Zno Films. Proceedings of the IEEE 1987 Ultrasonics Symposium.

[14] Vyun V.A., Umashev V.N., Yakovkin I.B. (1986). Yakovkin, Measurements of piezoelectric modulii of thin films. Sov. Phys..

[15] Caliendo C., Latino P.M. (2011). Characterization of Pt/Aln/Pt-Based Structures for High Temperature, Microwave Electroacoustic Devices Applications. Thin Solid Films.

[16] Verardi P., Craciun F. (2003). Acoustoelectric Probe for D33 Measurement on Piezoelectric Thin Films. Rev. Sci. Instrum..

[17] Tzou H.S., Fukuda T. (1992). Precision Sensors, Actuators and Systems.

[18] Adler E.L., Slaboszewicz J.K., Farnell G.W., Jen C.K. (1990). PC Software for SAW Propagation in Anisotropic Multilayers. IEEE Trans. Ultrason. Ferroelectr. Freq. Control.

[19] Colin K. (1998). Campbell, Surface Acoustic Wave Devices for Mobile and Wireless Communications.

[20] Santosh S.K. (2016). Surface Acoustic Wave Devices on Silicon Substrate Using Patterned and Thin Film Zno. Ph.D. Thesis.

[21] Wu P., Emanetoglu N.W., Tong X., Lu Y. Temperature Compensation of SAW in ZnO/SiO2 /Si Structure. Proceedings of the IEEE Ultrasonic Symposium.

[22] Le Brizoual L., Sarry F., Elmazria O., Alnot P., Ballandras S., Pastureaud T. (2008). GHz Frequency ZnO/Si SAW Device. IEEE Trans. Ultrason. Ferroelectr. Freq. Control.

[23] Aslam M.Z., Jeoti V., Karuppanan S., Malik A.F., Iqbal A. (2018). Malik and Asif Iqbal, FEM Analysis of Sezawa Mode SAW Sensor for VOC Based on CMOS Compatible AlN/SiO2/Si Multilayer Structure. Sensors.

[24] Caliendo C., Hamidullah M. (2017). Zero-Group-Velocity Acoustic Waveguides for High-Frequency Resonators. J. Phys. D Appl. Phys..

[25] Ahmadi M.T., Ismail R., Anwar S. (2017). Handbook of Research on Nanoelectronic Sensor Modeling and Applications.

[26] Ho C.K., Lindgren E.R., Rawlinson K.S., McGrath L.K., Wright J.L. (2003). Development of a Surface Acoustic Wave Sensor for In-Situ Monitoring of Volatile Organic Compounds. Sensors.

[27] Grate J.W., Abraham M.A. (1991). Solubility Interaction and Design of Chemically Selective Sorbent Coatings for Chemical Sensor and Arrays. Sens. Actuators B Chem..

[28] Ayela C., Heinrich S.M., Josse F., Dufour I. (2011). Resonant Microcantilevers for the Determination of the Loss Modulus of Thin Polymer Films. J. Microelectromech. Syst..

[29] Benedek I., Feldstein M.M. (2008). Technology of Pressure-Sensitive Adhesives and Products.

[30] Johnson S., Shanmuganantham T. (2014). Design and Analysis of SAW Based MEMS Gas Sensor for the Detection of Volatile Organic Gases. Carbon.

